# The Impact of Comorbidities on the Discontinuation of Antifibrotic Therapy in Patients with Idiopathic Pulmonary Fibrosis

**DOI:** 10.3390/ph18030411

**Published:** 2025-03-14

**Authors:** Stefano Kette, Nicolò Reccardini, Francesco Salton, Paola Confalonieri, Alessia Andrisano, Maria Chianese, Anna De Nes, Marta Maggisano, Alessandra Galantino, Salvatore Nicolosi, Marco Mari, Andrea Salotti, Darina Angoni, Maria Chernovsky, Michael Hughes, Marco Confalonieri, Lucrezia Mondini, Barbara Ruaro

**Affiliations:** 1 Pulmonology Unit, Department of Medical Surgical and Health Sciences, University of Trieste, Hospital of Cattinara, 34149 Trieste, Italy; 2 Pulmonology Unit, Department of Cardio-Thoracic Surgery, Health Integrated Agency of Friuli Venezia Giulia, 33100 Udine, Italy; 3 Division of Musculoskeletal and Dermatological Sciences, Faculty of Biology, Medicine and Health, The University of Manchester & Salford Royal NHS Foundation Trust, Manchester M6 8HD, UK

**Keywords:** idiopathic pulmonary fibrosis (IPF), comorbidities, antifibrotic therapy (AFT), nintedanib, pirfenidone, treatable traits

## Abstract

Idiopathic pulmonary fibrosis (IPF) is a chronic, progressive interstitial lung disease of unknown aetiology. Evidence on the progression of idiopathic pulmonary fibrosis (IPF) following the introduction of antifibrotic therapies still indicates a generally poor prognosis. IPF is associated with both respiratory and non-respiratory comorbidities, which can worsen symptoms and impact overall survival. **Background/Objectives**: The study aimed to investigate the effect of these comorbidities on the early and permanent discontinuation of pirfenidone or nintedanib in IPF patients. **Methods**: In this single-centre retrospective study, 101 patients diagnosed with IPF according to ATS/ERS/JRS/ALAT guidelines were treated with AFT. Clinical data were collected at 12 months prior to and up to 24 months following treatment initiation, including age, gender, smoking history, and the presence of respiratory and non-respiratory comorbidities. **Results:** The data showed that 21 patients (20.8%) discontinued treatment within the first 12 months. Additionally, pre-treatment comorbidities were not statistically correlated with the suspension of antifibrotic treatment. Among the overall cohort, 77 patients (76.2%) had at least one comorbidity and 27 (26.7%) had three or more comorbidities. Notably, 24 (23.8%) had respiratory comorbidities, while 75 (74.3%) had non-respiratory comorbidities. **Conclusions:** This real-life study emphasises the complexities involved in managing IPF, particularly regarding adherence to treatment when significant comorbidities are present. The evidence suggests that in patients with IPF, pre-treatment respiratory or non-respiratory conditions do not affect AFT discontinuation.

## 1. Introduction

IPF accounts for >30% of all interstitial lung disease (ILD) cases [1]. It is a chronic, progressive, and specific form of ILD associated with the histopathological and/or radiological pattern of usual interstitial pneumonia (UIP). The diagnosis requires identification of a distinct UIP pattern on high-resolution computed tomography (HRCT) when no clear aetiology, connective tissue disease, or other known causes of ILD are present [2,3,4,5,6]. Other patterns, including probable UIP, require histological confirmation [7].

Epidemiological data demonstrate that the worldwide prevalence of IPF ranges from 0.33 to 4.51 per 10,000 persons and the global incidence ranges from 0.09 to 1.30 per 10,000 [8,9]. Despite some therapeutic options, the average survival without treatment remains limited, between 3 and 5 years post-diagnosis [6,7,10].

IPF should be considered in patients over 60, predominantly male, presenting with unexplained bilateral pulmonary fibrosis on chest radiography (CXR) or CT scans. Symptoms include dyspnoea, fatigue, and dry cough, with bibasilar crackles on examination. A history of smoking and environmental or occupational exposure is often reported [11,12,13,14].

In October 2014, the US Food and Drug Administration (FDA) approved two antifibrotic drugs, pirfenidone and nintedanib, which represent a milestone in the treatment of IPF and are currently used in its management [15,16,17,18,19]. Prior to that, no evidence-based and effective treatments were available. The management of IPF mainly relied on the use of immunosuppressants and corticosteroids, until the PANTHER trial was published, which provided strong evidence against the use of these drugs [20,21].

Different classes of drugs were also examined. Based on the potential prothrombotic state associated with IPF, therapeutic anticoagulation with warfarin was proposed [22,23]. However, the trial was halted due to higher mortality in the warfarin group [24].

Additionally, no effects on the specified outcomes were observed in trials of endothelin receptor antagonists, cyclophosphamide, and etanercept [25,26,27,28].

Based on the altered microbiota, antibiotic trials with trimethoprim-sulfamethoxazole or doxycycline showed no impact on hospitalisation time or mortality compared to usual care [29,30].

In contrast, the advent of pirfenidone and nintedanib demonstrated efficacy in randomised clinical trials (RCTs) by slowing the decline in Forced Vital Capacity (FVC) [19,31].

Nevertheless, limited effects on symptom burden, lung diffusing capacity for carbon monoxide (DLCO), reduction in lung fibrosis assessed via high-resolution computed tomography, walk test outcomes, and health-related quality of life were proven [16,19,32].

These two drugs are also associated with several side effects, mainly gastrointestinal (GI) issues, including nausea, diarrhoea, and dyspepsia, as well as skin-related conditions, which may lead to treatment discontinuation or dose reduction [33]. Notably, high monounsaturated fatty acid diets have been found to result in fewer pirfenidone-related gastrointestinal side effects compared to high saturated fatty acid diets [34].

In light of these treatment limitations, 60 to 70% of IPF deaths result from the progression of lung disease, with the remaining cases attributed to pulmonary and extra-pulmonary comorbidities, which are increasingly recognised as impacting survival and quality of life (QoL) in IPF [35,36,37,38]. These are common and often observed in patients with IPF, with some sharing risk factors like tobacco exposure, while others, such as PH, may result from IPF progression [38].

Respiratory disorders include chronic obstructive pulmonary disease (COPD) (37%), lung cancer (3%, but some studies report up to 20.4% over 10 years), pulmonary embolism (PE) (1.60%), pulmonary hypertension (PH) (30–50% of patients with advanced IPF develop PH), and obstructive sleep apnoea (OSA) (60%) [39,40].

Additionally, non-respiratory conditions in IPF include gastroesophageal reflux disease (GERD) (30% to over 80%), dyslipidaemia (13%), cardiovascular disease (CVD) (14–63%), diabetes mellitus (10% and 33%), and anxiety/depression (20 to 49%) [41,42]. Among these lung cancers, PH and CVDs are particularly impactful, significantly influencing prognosis and survival [43,44,45,46].

The influence of comorbidities on the discontinuation or dose reduction of antifibrotic drugs has not been clearly defined. Available data on their impact are limited and occasionally contradictory [47,48]. Some studies suggested comorbidities may potentially increase antifibrotic drug toxicity, prompting treatment discontinuation. Others noted that patients can remain on therapy if comorbidities are properly managed [49,50,51].

The objective of this study was to determine whether any specific comorbidity was particularly associated with treatment discontinuation at our tertiary referral centre for interstitial lung diseases.

## 2. Results

The study cohort comprised 101 patients with IPF on antifibrotic treatment, of whom 62 (61.4%) were male, with median [interquartile range {IQR}] age at baseline 73.0 (11.0) years and median [IQR] age at disease diagnosis 70.0 [13.0] years (Table 1). Eighty-four (83.2%) diagnoses were based on the HRCT pattern and 17 (16.8%) on the histological pattern (Table 1). Forty-eight (47.5%) patients were treated with pirfenidone and 53 (52.5%) with nintedanib. Overall, 21 (20.8%) patients had their treatment suspended during the first 12 months of therapy: six (12.5%) in the pirfenidone subgroup and 15 (28.3%) in the nintedanib subgroup.

**Figure A1:**
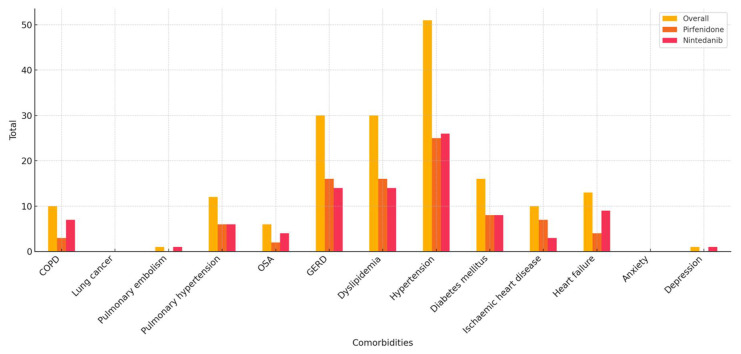
Prevalence of respiratory and non-respiratory comorbidities before starting antifibrotic treatment.

**Figure A2:**
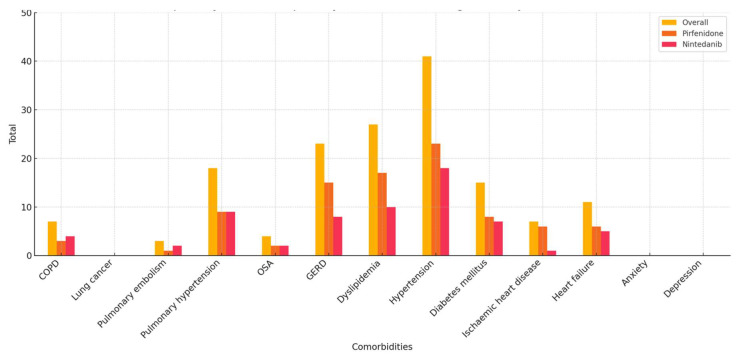
Prevalence of respiratory and non-respiratory comorbidities during the first year of antifibrotic treatment.

**Figure A3:**
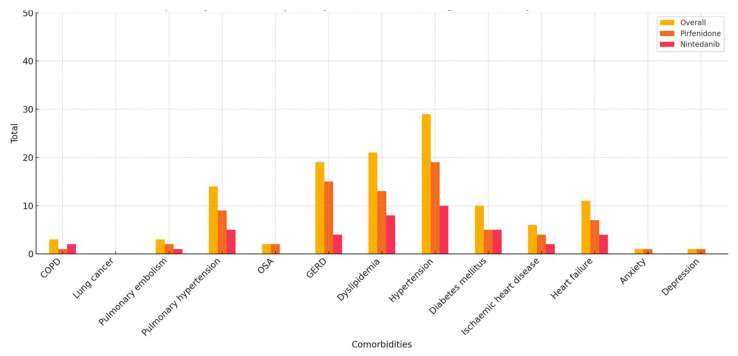
Prevalence of respiratory and non-respiratory comorbidities during the second year of antifibrotic treatment.

Pre-treatment, in the entire cohort, 77 (76.2%) patients had at least one comorbidity and 27 (26.7%) had ≥3 comorbidities; 24 (23.8%) had at least one respiratory comorbidity and 75 (74.3%) had at least one non-respiratory comorbidity. PH (12 [11.9%]) and systemic hypertension (51 [50.5%]) were the most common respiratory and non-respiratory comorbidities, respectively. The prevalence of comorbidities (pre-treatment, first year, and second year) and their stratification by treatment protocol are described in Table 2 and Figure A1, Figure A2 and Figure A3.

In univariate analysis, no pre-treatment comorbidity correlated significantly with antifibrotic treatment suspension during the first 12 months of therapy. Consistent results were obtained when stratifying by antifibrotic drug (Table 3). Similarly, age, sex, antifibrotic drug, presence of ≥3 comorbidities, presence of ≥1 respiratory comorbidity, and presence of ≥1 non-respiratory comorbidity did not significantly correlate to treatment suspension (Table S1).

## 3. Discussion

The findings from this retrospective study provide valuable insights into the evolution of comorbidity prevalence in patients with IPF undergoing AFT in a tertiary care centre for interstitial lung diseases [48,52,53,54,55,56,57,58,59,60,61]. With a cohort of 101 patients, the demographic and clinical profiles shed light on the complexities associated with managing this progressive lung disease. The main results of this study demonstrate that the pre-treatment comorbidities did not show a significant correlation with antifibrotic treatment suspension. During the first 12 months, 21 patients discontinued treatment. Pirfenidone was linked to fewer withdrawals (six patients—12.5%), mainly due to fewer side effects compared to nintedanib (15 patients—28.3%). This is consistent with existing literature [47,50,62].

### 3.1. Demographic and Clinical Characteristics

A comparison of the demographics of patients treated with either antifibrotic treatment revealed that the study population predominantly consisted of older males, with a median age of 73 years at baseline and a median age of 70 years at diagnosis. This aligns with existing literature, which consistently identifies IPF as a disease that primarily affects older adults, particularly males [48,52,53,54,55,56,57,58,59,60,61,63,64,65,66,67,68].

Notably, the treatment distribution between the two groups was balanced, with 47.5% and 52.5% of patients receiving each drug, respectively.

The pirfenidone group contained a slightly greater percentage of females (45.8%) compared to the nintedanib group (32.1%), and patients in the pirfenidone group were younger on average, with a mean age of 69.5 years versus 75 years in the nintedanib group.

The majority of IPF diagnoses (83.2%) relied on High-Resolution Chest CT (HRCT), whereas histological confirmation was required only in 16.8% of the patients. This underlines the importance of recognising the characteristic pattern of IPF on HRCT to minimise the need for invasive lung biopsy [53].

Different degrees of functional impairment across the study population in the baseline PFT were observed. The median predicted FVC was 77%, with slightly higher values in the pirfenidone group (81%) compared to the nintedanib group (74.5%). In contrast, the DLCO was lower in the pirfenidone group compared to the nintedanib group (41% vs. 48%). Pirfenidone has been shown to slow down disease progression by positively affecting the decline of FVC (which decreases less quickly) [29,30,31,32,67]. This leads to better outcomes. As seen with pirfenidone, patients treated with nintedanib have shown an impaired decrease in FVC, which has helped to slow down disease progression [37,38,39,67,68,69,70,71,72,73,74]. Although they have shown efficiency, these two drugs, whether taken in isolation or with other therapies, did not prevent fatal outcomes in IPF patients [67,75,76,77,78,79,80,81,82,83,84].

### 3.2. Treatment Patterns and Discontinuation Rates

Pirfenidone and nintedanib are the only authorised AFTs in the treatment of IPF. Based on the results of two phase-III clinical trials, both medications demonstrated effectiveness in slowing disease progression [16,19]. Nintedanib is a receptor antagonist that interacts with several tyrosine kinases, including the receptors for vascular endothelial growth factor (VEGF), fibroblast growth factor (FGF), and platelet-derived growth factor (PDGF) [19,54]. Pirfenidone is an antifibrotic agent that inhibits collagen synthesis stimulated by transforming growth factor beta (TGF-β) and additionally suppresses fibroblast proliferation in vitro [55].

Despite these outcomes, several side effects have been reported [16,19,47]. These include GI symptoms (nausea, dyspepsia, vomiting, and anorexia), fatigue, dizziness, and skin reactions (rashes and photosensitivity), all of which ultimately led to therapy discontinuation [53].

Our finding of a 20.8% overall treatment suspension rate within the first 12 months is concerning. This raises important questions about the tolerability and side-effect profiles of AFT. Previous literature reported similar discontinuation rates, with adverse events and medication tolerance as crucial factors influencing treatment adherence [16,19,53].

The discontinuation rate varies across studies and is highly dependent on the study design, ranging from 15% in the two main pirfenidone RCTs to 54.4% in a recent multicentre observational study [16,50,53]. In the INPULSIS-1 and INPULSIS-2 trials, the rates were 25.2% and 23.7% for nintedanib, respectively, while the same observational study reported a 64% discontinuation rate [19]. Our results showed 15 patients (28.3%) in the nintedanib subgroup and six patients (12.5%) in the pirfenidone subgroup discontinuing treatment.

Furthermore, other factors, such as patient preferences and physician-patient communication, must also be taken into consideration, as they can significantly impact treatment adherence [67,79,80,81,82,83,84]. These findings underscore the importance of assessing the patient’s overall health context when considering treatment strategies.

The correct management of these complications is crucial to maintaining treatment adherence in patients treated with AFT, slowing disease progression, and ultimately improving prognosis.

### 3.3. Comorbidities and Their Impact

The high prevalence of comorbidities in our cohort is particularly noteworthy: 76.2% of patients had at least one comorbidity, and multimorbidity was common, considering that 26.7% had three or more. Previous studies have similarly reported high rates of comorbid conditions in IPF patients, with CVDs, diabetes mellitus, and PH frequently cited as common comorbidities, particularly among older IPF populations [16,56,57]. Notably, the higher number of comorbidities was associated with increased mortality. This is particularly concerning given the frequent occurrence of multimorbidity in IPF patients [42,56,58].

The types of comorbidities present in this cohort warrant further discussion. Supine hypertension (SH) (50.5%) and PH (11.9%) were the most prevalent comorbidities, emphasising the need for comprehensive cardiovascular assessments in individuals with IPF. As a single-centre retrospective study, variability in comorbidity prevalence and incidence may be observed when compared to studies with different designs and direct comparisons can be challenging [38,41,42,59]. For instance, in the INSIGHTS-IPF registry, which included over 500 patients from 19 centres in a prospective observational study [38], the most frequently observed comorbidities were SH, similar to our findings, coronary heart disease, and diabetes mellitus.

Additionally, no cases of lung cancer were reported at baseline or during the 24-month follow-up in our cohort, despite the majority of our patients having a history of smoking (61.4%). Patients often report exposure to inhaled dust, asbestos, metal, and wood particles, with air pollution also linked to increased IPF incidence and mortality [12,60,61,63,64,65,66,67].

Existing literature reports that the risk of lung cancer in patients with IPF is higher compared to the general population. Up to 20% of patients with IPF develop lung cancer [63]. In patients with both emphysema and fibrosis, the risk of lung cancer increases to 12%. Even interstitial lung abnormalities (ILA) showed an increased risk of potential cancer in the National Lung Screening Trial. This risk appears to affect all histological subtypes of lung cancer [61].

Lung cancer in IPF differs from non-fibrotic cancers, with smokers typically developing adenocarcinoma, while squamous cell carcinoma, often peripheral and adjacent to usual interstitial pneumonia, was found to be more common in IPF patients [67,79,80,81,82,83,84].

In our cohort, COPD was present in almost 10% of patients. The prevalence of this disease was in line with most previous publications, with percentage values below those reported in the literature [56,63]. The suspension rate in this group was 20% overall, with 33.3% in the pirfenidone subgroup and 14.3% in the nintedanib subgroup. At the 12-month mark, two patients with COPD discontinued AFT; however, no statistically significant correlation with treatment discontinuation was found (*p* > 0.05).

Similarly for PE, while a meta-analysis demonstrated a twofold increase in the risk of venous thromboembolism (VTE) in IPF patients compared to the general population [64], no treatment discontinuation was observed in our patients with PE across both treatment groups. This could denote a minimal impact of PE on treatment adherence.

Interestingly, a higher suspension rate (33.3% overall; 50% for the nintedanib cohort) was depicted in patients with OSA, compared to 20.0% of those without OSA, but the limited number of events reasonably reduced the power to detect statistically significant differences. Previous studies highlighted that nocturnal oxygen desaturation is associated with a worse prognosis in IPF [65,66]. Several studies reported that Sleep Breathing Disorders (SBD) are associated with a worse prognosis in terms of mortality and clinical progression of the disease [67,79,80,81,83,84].

When considering extra-pulmonary conditions, in the literature the prevalence of GERD is high, being reported to affect up to 87% of patients, of whom only half 47% are symptomatic [67]. However, there is a high variability in the prevalence of GERD and reflux symptoms, ranging from 0% to 94%, depending on the study design and assessment method [41,56,68,69]. Our data were slightly lower, as we only considered symptomatic GERD, for which treatment had been initiated by the clinician. In our cohort, GERD was present in 29.7% of patients.

A proposed mechanism of injury in IPF related to GERD suggests that microaspiration of gastric contents may contribute to ongoing alveolar epithelial injury, subsequently promoting fibrosis and potentially accelerating IPF progression [15,70]. Despite this association, the efficacy of anti-reflux therapies in IPF remains uncertain.

While some observational and retrospective studies suggested that antacid treatment and GERD therapy in IPF patients may potentially reduce the rate of IPF progression and improve survival outcomes [69,71], recent RCTs and post hoc analyses did not demonstrate significant benefits [72,73]. Consequently, the routine use of anti-reflux treatment in IPF patients is not currently recommended [48].

In a recent monocentric study conducted by Hyldgaard et al., involving 121 patients, CVD, depression, and SH were identified as the most common comorbidities among patients with IPF [41]. Similarly, another study reported that SH was present in 39% of patients with unclassifiable interstitial lung disease [74]. Consistently, SH was also a common finding in our cohort, affecting 50.5% of patients overall.

However, unlike Hyldgaard et al.’s findings, we observed a lower prevalence of depression in our cohort. Only one patient had depression before starting AFT, and another developed anxiety in the second year. This discrepancy may be due to sample size, as larger cohorts are more likely to include these conditions [75]. Both anxiety and depression have a significant impact on health-related quality of life [76,77,78,79,80,81,82,83,84].

Other comorbidities, including IHD, dyslipidaemia, and diabetes mellitus, did not demonstrate a significant correlation with treatment discontinuation in the overall cohort or within either AFT subgroup. For instance, the suspension rate for SH remained similar between those with and without the condition (21.6% vs. 20.0%). Dyslipidaemia had an overall suspension rate of 26.7%, with no notable difference between the two treatment groups (*p* = 0.344). Likewise, diabetes mellitus had a low suspension rate of 12.5%, with rates of 25.0% in the pirfenidone group and 0.0% in the nintedanib group, showing no substantial difference (*p* = 0.373 and *p* = 0.054, respectively). Similar findings were reported in a South Korean nationwide cohort study [39].

The absence of significant relationships between comorbidities and treatment outcomes suggests that, while prevalent, comorbid conditions may not directly dictate treatment decisions or outcomes in the short term. This finding is consistent with existing literature, which posits that the impact of comorbidities on treatment efficacy may be more nuanced and context-dependent [67,79,80,81,82,83,84]. This points to the necessity of further research to identify additional predictors of treatment adherence, such as psychological factors or socioeconomic status. In addition, it also remains unclear to what extent comorbidities can influence IPF progression in addition to their own direct adverse health effects [77].

### 3.4. Treatable Traits

Treatable traits refer to specific characteristics or comorbidities associated with a patient’s condition that can be targeted for intervention. In the context of IPF, recognising these traits can enhance the overall management of the disease, improve patients’ quality of life, and potentially alter disease progression.

Treatable traits represent a patient-centred approach that focuses on identifying and addressing specific modifiable factors. A model of care has already been introduced for other lung conditions, such as asthma and bronchiectasis [78,79,80,81]. Even in the latest GOLD 2025 report for COPD, a strategy based on these has been proposed [82].

This is conventionally divided into pulmonary, extrapulmonary, aetiological, and behavioural/lifestyle domains, all of which deserve individual attention and treatment when present [78]. Pulmonary causes include progressive fibrosis, eosinophilic or neutrophilic inflammation, acute exacerbations, infections (whether acute, chronic, or recurrent), chronic respiratory failure, intractable chronic cough, and structural issues like emphysema or obstructive ventilatory defects.

In addition, common comorbidities such as gastroesophageal reflux disease (GERD), obstructive sleep apnoea (OSA), pulmonary hypertension (PH), congestive heart failure (CHF), lung cancer, diabetes, osteoporosis or osteopenia, pulmonary embolism (PE), and conditions like obesity, cachexia, malnutrition, and frailty can significantly impact disease outcomes. Moreover, lifestyle factors are also considered treatable traits. Smoking cessation is crucial for IPF patients, as continued smoking can exacerbate lung damage. Similarly, encouraging physical activity and pulmonary rehabilitation can improve exercise tolerance, reduce symptoms, and enhance quality of life. Therefore, multidisciplinary strategies are required [80,81,82,83,84,85,86,87].

Furthermore, anxiety and depression are integral components of the treatable traits paradigm. These can significantly reduce patients’ quality of life. For instance, the Hospital Anxiety and Depression Scale (HADS) is a widely used scale that measures anxiety and depression levels, providing separate scores for each [75].

Furthermore, advancements in personalised medicine are allowing for more tailored approaches to treatment. For example, targeted therapies that consider specific genetic or molecular characteristics of a patient’s IPF may emerge, providing more effective management options.

In summary, the relevance of treatable traits in IPF lies in their potential to provide a more holistic approach to patient care. By identifying and addressing comorbidities, lifestyle factors, and psychological challenges, healthcare providers can enhance the quality of life for patients and potentially influence the progression of the disease. This comprehensive management strategy underscores the importance of viewing IPF not just as a standalone condition but as part of a broader context of patient health and well-being.

### 3.5. Limitations

While this study provides important insights, it has some limitations. First, this was a retrospective design and therefore selection bias may have been introduced. Treatment practices, patient characteristics, and comorbidity prevalence may differ across centres, making it difficult to extrapolate these results to broader populations

Prospective studies are necessary to assess the impact of comorbidities on AFT discontinuation. Secondly, the sample size was small, though the two groups were equally balanced, with 48 patients receiving pirfenidone and 53 receiving nintedanib. This may have limited the ability to detect significant associations, particularly for less common comorbidities.

Furthermore, the timing of initiating AFT and the choice between nintedanib or pirfenidone is ultimately up to the physician. No current guidelines have been released on this topic so far.

Further research with larger, well-controlled cohorts and standardised diagnostic criteria is needed to explore the complex interplay between comorbidities, treatment adherence, and disease outcomes.

### 3.6. Future Directions

Despite all these limitations, we strongly believe that the management of IPF patients remains a complex challenge for clinicians, especially due to the high prevalence of comorbidities and treatment adherence.

In current clinical practice, genetic testing for prognosis or treatment guidance in IPF is limited, with no specific recommendations in the latest guidelines [48]. In the coming years, Next Generation Sequencing (NGS) may help identify genetic variants linked to disease progression, treatment response, and comorbidities. Integrating NGS with biomarkers and clinical data could enhance personalised treatment and prognostic models. First and foremost, there is a need for robust long-term studies to evaluate the efficacy and safety of antifibrotic therapies over extended periods. While current data suggest that these drugs can slow the decline in lung function, their long-term impact on quality of life, survival rates, and the overall burden of disease remains to be fully elucidated. Research should aim to identify not only the benefits of prolonged treatment but also any potential side effects that may emerge over time. Another promising area for future research is the exploration of combination therapies. Given the multifactorial nature of IPF, combining antifibrotic drugs with other therapeutic modalities, such as anti-inflammatory agents or immunomodulators, could offer a synergistic effect that enhances patient outcomes. Investigating the mechanisms underlying fibrosis and inflammation in IPF may lead to the identification of novel targets for therapy, thereby broadening the scope of treatment options available. Additionally, the development of personalised medicine approaches in treating IPF could revolutionise patient care. Genetic and biomarker studies can help identify subgroups of patients who may respond better to specific antifibrotic therapies. Understanding the molecular and genetic underpinnings of IPF can lead to tailored treatment plans that optimise therapeutic efficacy and minimise adverse effects, ultimately improving patient outcomes. Furthermore, research should not overlook the role of patient-reported outcomes and their importance in understanding the overall impact of IPF and its treatments on daily life. Incorporating qualitative research methods to capture the lived experiences of patients can provide valuable insights into how antifibrotic drugs affect their quality of life, emotional well-being, and social functioning. Lastly, global disparities in access to antifibrotic treatments must be addressed. Future research should explore ways to enhance access to these critical therapies, particularly in low- and middle-income countries where the burden of lung diseases, including IPF, is often underrecognised and undertreated. Collaborative efforts among researchers, healthcare providers, and policymakers are essential to ensure that all patients with IPF can benefit from advancements in treatment. In conclusion, the future of IPF research, particularly in the realm of antifibrotic therapies, holds great promise. By focusing on long-term efficacy, combination therapies, personalised medicine, patient-centred outcomes, and equitable access, researchers can pave the way for more effective management strategies. As we continue to unravel the complexities of IPF, the hope is that these efforts will lead to improved quality of life and survival for those affected by this challenging disease. Finally, larger, multicentre, and prospective studies are essential to validate the results of this study. These studies would provide valuable insights into how comorbidities impact treatment decisions.

## 4. Materials and Methods

### 4.1. Study Design and Population

This retrospective study included all adults (aged 18 years or older) diagnosed with IPF who initiated antifibrotic treatment with either pirfenidone or nintedanib between 2011 and 2024 at the Department of Pulmonology, University Hospital of Cattinara, Trieste, Italy. Patients were excluded from the enrolment if they had inadequate clinical information, lacked diagnostic imaging or histological data supporting the diagnosis, had a diagnosis of connective tissue diseases or a more convincing alternative diagnosis, or received inappropriate treatment protocols.

IPF diagnosis was established in accordance with the 2018 ATS/ERS/JRS/ALAT Clinical Practice Guideline [7]. The evaluation of interstitial lung disease (ILD) at our centre relied on HRCT and/or lung tissue biopsy. All diagnostic assessments were performed by pulmonologists, radiologists, and pathologists with over 10 years of experience in lung imaging and histology.

The initiation of therapy marked the baseline, where all included patients began either pirfenidone or nintedanib treatment. For pirfenidone, any daily dose achieved following the recommended 14-day titration period was accepted as the “starting dose.” According to EMA/AIFA guidelines, the full dose was defined as three 267 mg capsules taken three times daily (2403 mg/day), while adjusted doses consisted of one to two capsules (267 mg–534 mg) taken two to three times daily. For nintedanib, the “starting dose” was defined as any daily dosage prescribed by the referring physician in accordance with the drug’s data sheet. Per EMA/AIFA guidelines, the full dose was defined as two 150 mg capsules twice daily, while the adjusted dose consisted of two 100 mg capsules twice daily. Permanent discontinuation of therapy was classified as treatment suspension. All modifications to treatment protocols were discussed and approved by specialists prior to implementation.

### 4.2. Aim of the Study

This study aims to describe the evolution of the prevalence of respiratory and non-respiratory comorbidities before and during antifibrotic treatment with pirfenidone and nintedanib in patients with IPF. Secondly, it proposes to investigate a possible correlation between pre-treatment comorbidities and early and permanent discontinuation of therapy.

### 4.3. Data Collection

Clinical data were collected retrospectively from 12 months before baseline up to 24 months after. The following clinical parameters were recorded: sex, age at baseline, age at diagnosis, smoking history, respiratory and non-respiratory comorbidities (including COPD, lung cancer, PE, PH, OSA, GERD, dyslipidaemia, SH, diabetes mellitus, ischemic heart disease (IHD), CHF, anxiety, and depression), pirfenidone and nintedanib therapeutic doses, changes in treatment protocols, and time to treatment adjustments.

Any relevant comorbidity documented during clinical assessments within the 12 months prior to baseline was classified as “pre-treatment”. Comorbidities identified in assessments conducted during the first 12 months post-baseline were categorised as “first year”, while those recorded during the subsequent 12 months were classified as “second year”.

### 4.4. Statistical Analysis

Data were summarised using the mean (standard deviation [SD]) or median (interquartile range [IQR]) for continuous variables, and absolute and relative frequencies (percentages) for categorical variables. The normality of continuous variables was evaluated using the Shapiro–Wilk test. When describing the development of comorbidities during antifibrotic treatment, only patients undergoing at least 4 and 9 months of therapy were included in the analysis of the first and second year, respectively. Differences between groups were analysed using the chi-square test for categorical variables and the Mann–Whitney U test for continuous variables. A two-tailed *p*-value < 0.05 was considered statistically significant. All analyses were conducted using JASP software (version 0.19.1, Eric-Jan Wagenmakers, Department of Psychological Methods, University of AmsterdamNieuwe Achtergracht 129B, Amsterdam, The Netherlands, E.U.) and data visualisation was performed using ChatGPT-4o (OpenAI © 2015–2025; San Francisco, CA, USA).

## 5. Conclusions

IPF is a complex and challenging disease to manage, characterised by a poor prognosis. To date, therapeutic interventions have primarily aimed at mitigating the progression of lung function decline. The use of approved antifibrotic agents is often associated with significant adverse effects, which may lead to treatment discontinuation. The comorbidities associated with IPF in typically elderly patients have been hypothesised to influence the permanent discontinuation of antifibrotic therapy. Nonetheless, our real-life study suggests that comorbidities do not directly influence the decision to discontinue treatment, even when they manifest at an early stage, although they may contribute to the overall deterioration of health status; further research is needed to identify the factors contributing to treatment discontinuation, with the ultimate goal of enhancing the quality of care and clinical outcomes for patients with IPF. However, our results confirm that patients with IPF and comorbidities, either due to a respiratory or non-respiratory condition, have more severe disease with a worse prognosis. Optimising the management of comorbid conditions through or enhancing a multifaceted approach to patient care and assessing the patient’s overall health context when considering treatment strategies could potentially improve both quality of life and survival.

## Figures and Tables

**Table 1:** Baseline characteristics of the study population.

		Overall	Pirfenidone	Nintedanib
		n = 101	n = 48	n = 53
Age (years)		73.0 (11.0)	69.5 (11.0)	75.0 (9.0)
Disease onset (years)		70.0 (11.0)	67.0 (9.8)	73.0 (9.0)
Gender				
	Males	62 (61.4)	26 (54.2)	36 (67.9)
	Females	39 (38.6)	22 (45.8)	17 (32.1)
Ever-smoker		62 (61.4)	32 (66.7)	30 (56.6)
Diagnosis				
	HRCT	84 (83.2)	41 (85.4)	43 (81.1)
	Histological	17 (16.8)	7 (14.6)	10 (18.9)
Starting dose (mg/die)			2403.0 (0.0)	300.0 (0.0)
Pulmonary function tests				
	FEV1% predicted	83.5 (28.0)	84.0 (35.0)	81.0 (27.0)
	FVC % predicted	77.0 (28.3)	81.0 (32.5)	74.5 (22.8)
	DLCO % predicted	43.0 (27.0)	41.0 (23.0)	48.0 (27.5)

Data are presented as No. (%) or median (IQR), unless otherwise stated. Abbreviations: HRCT, high-resolution computed tomography; FEV1, forced expiratory volume in 1 s; FVC, forced vital capacity; DLCO, diffusing lung capacity for carbon monoxide.

**Table 2:** Prevalence of respiratory and non-respiratory comorbidities before starting antifibrotic treatment, during the first and second years of therapy.

		Pre-Treatment			First Year			Second Year		
		Overall	Pirfenidone	Nintedanib	Overall	Pirfenidone	Nintedanib	Overall	Pirfenidone	Nintedanib
		n = 101	n = 48	n = 53	n = 86	n = 45	n = 41	n = 75	n = 43	n = 32
Respiratory										
	COPD	10 (9.9)	3 (6.3)	7 (13.2)	7 (8.1)	3 (6.7)	4 (9.8)	3 (4.0)	1 (2.3)	2 (6.3)
	Lung cancer	0 (0.0)	0 (0.0)	0 (0.0)	0 (0.0)	0 (0.0)	0 (0.0)	0 (0.0)	0 (0.0)	0 (0.0)
	Pulmonary embolism	1 (1.0)	0 (0.0)	1 (1.9)	3 (3.5)	1 (2.2)	2 (4.9)	3 (4.0)	2 (4.7)	1 (3.1)
	Pulmonary hypertension	12 (11.9)	6 (12.5)	6 (11.3)	18 (20.9)	9 (20.0)	9 (22.0)	14 (18.7)	9 (20.9)	5 (15.6)
	OSA	6 (5.9)	2 (4.2)	4 (7.5)	4 (4.7)	2 (4.4)	2 (4.9)	2 (2.7)	2 (4.7)	0 (0.0)
Non-respiratory										
	GERD	30 (29.7)	16 (33.3)	14 (26.4)	23 (26.7)	15 (33.3)	8 (19.5)	19 (25.3)	15 (34.9)	4 (12.5)
	Dyslipidemia	30 (29.7)	16 (33.3)	14 (26.4)	27 (31.4)	17 (37.8)	10 (24.4)	21 (28.0)	13 (30.2)	8 (25.0)
	Hypertension	51 (50.5)	25 (52.1)	26 (49.1)	41 (47.7)	23 (51.1)	18 (43.9)	29 (38.7)	19 (44.2)	10 (31.3)
	Diabetes mellitus	16 (15.8)	8 (16.7)	8 (15.1)	15 (17.4)	8 (17.8)	7 (17.1)	10 (13.3)	5 (11.6)	5 (15.6)
	Ischaemic heart disease	10 (9.9)	7 (14.6)	3 (5.7)	7 (8.1)	6 (13.3)	1 (2.4)	6 (8.0)	4 (9.3)	2 (6.3)
	Heart failure	3 (12.9)	4 (8.3)	9 (17.0)	11 (12.8)	6 (13.3)	5 (12.2)	11 (14.7)	7 (16.3)	4 (12.5)
	Anxiety	0 (0.0)	0 (0.0)	0 (0.0)	0 (0.0)	0 (0.0)	0 (0.0)	1 (1.3)	1 (2.3)	0 (0.0)
	Depression	1 (1.0)	0 (0.0)	1 (1.9)	0 (0.0)	0 (0.0)	0 (0.0)	1 (1.3)	1 (2.3)	0 (0.0)

Data are presented as No. (%), unless otherwise stated. When presenting data during the first year of treatment, only patients undergoing therapy for at least 4 months were included. When presenting data during the second year of treatment, only patients undergoing therapy for at least 9 months were included. Abbreviations: COPD, chronic obstructive pulmonary disease; OSA, obstructive sleep apnoea; GERD, gastroesophageal reflux disease.

**Table 3:** Correlation between baseline comorbidities and treatment suspension during the first year in the entire cohort and according to the antifibrotic drug ^#^.

		Overall		Pirfenidone		Nintedanib	
		n = 101	*p*-Value	n = 48	*p*-Value	n = 53	*p*-Value
COPD							
	Yes	2 (20.0)	0.948	1 (33.3)	0.260	1 (14.3)	0.377
	No	19 (20.9)		5 (11.1)		14 (30.4)	
Pulmonary embolism							
	Yes	0 (0.0)	0.607	0 (0.0)	¶	0 (0.0)	0.526
	No	21 (21.0)		6 (12.5)		15 (28.8)	
Pulmonary hypertension							
	Yes	1 (8.3)	0.257	0 (0.0)	0.322	1 (16.7)	0.502
	No	20 (22.5)		6 (14.3)		14 (29.8)	
OSA							
	Yes	2 (33.3)	0.435	0 (0.0)	0.585	2 (50.0)	0.316
	No	19 (20.0)		6 (13.0)		13 (26.5)	
GERD							
	Yes	7 (23.3)	0.682	2 (12.5)	1.000	5 (35.7)	0.473
	No	14 (19.7)		4 (12.5)		10 (25.6)	
Dyslipidaemia							
	Yes	8 (26.7)	0.344	3 (18.8)	0.355	5 (35.7)	0.473
	No	13 (18.3)		3 (9.4)		10 (25.6)	
Hypertension							
	Yes	11 (21.6)	0.846	3 (12.0)	0.913	8 (30.8)	0.696
	No	10 (20.0)		3 (13.0)		7 (25.9)	
Diabetes mellitus							
	Yes	2 (12.5)	0.373	2 (25.0)	0.242	0 (0.0)	0.054
	No	19 (22.4)		4 (10.0)		15 (33.3)	
Ischaemic heart disease							
	Yes	2 (20.0)	0.948	1 (14.3)	0.877	1 (33.3)	0.842
	No	19 (20.9)		5 (12.2)		14 (28.0)	
Heart failure							
	Yes	2 (15.4)	0.607	1 (25.0)	0.430	1 (11.1)	0.209
	No	19 (21.6)		5 (11.4)		14 (31.8)	
Depression							
	Yes	1 (100.0)	0.05	0 (0.0)	¶	1 (100.0)	0.108
	No	20 (20.0)		6 (12.5)		14 (26.9)	

Data are presented as No. (%), unless otherwise stated. Abbreviations: COPD, chronic obstructive pulmonary disease; OSA, obstructive sleep apnoea; GERD, gastroesophageal reflux disease. ^#^ Lung cancer and anxiety were omitted due to the absence of events; ¶ Chi-squared could not be calculated due to the absence of relevant events.

## Data Availability

All the data are available upon reasonable request to the corresponding author.

## Supplementary Materials

The following supporting information can be downloaded at: https://www.mdpi.com/article/10.3390/ph18030411/s1. Table S1. Correlation between baseline characteristics and treatment suspension during the first year in the entire cohort and according to the anti-fibrotic drug.

## Abbreviations

The following abbreviations are used in this manuscript:

6MWD	6-Minute Walking Distance
AE	Adverse Effects
AFT	Antifibrotic Therapy
ALAT	Asociación Latinoamericana De Tórax
ALT	Alanine Aminotransferase
AST	Aspartate Aminotransferase
ATS	American Thoracic Society
COPD	Chronic Pulmonary Obstructive Disease
CVD	Cardiovascular Diseases
CXR	Chest Radiography
DLCO	Diffusing Lung Capacity for Carbon Monoxide
ERS	European Respiratory Society
FVC	Forced Vital Capacity
GERD	Gastroesophageal Reflux Disease
GI	Gastrointestinal
HRTC	High-Resolution Computed Tomography
IHD	Ischemic Heart Disease
ILA	Interstitial Lung Abnormalities
ILD	Interstitial Lung Disease
IPF	Idiopathic Pulmonary Fibrosis
IQR	Interquartile Range
JRS	Japanese Respiratory Society
OSA	Obstructive Sleep Apnoea
PE	Pulmonary Embolism
PH	Pulmonary Hypertension
QoL	Quality of Life
RCT	Randomised Clinical Trial
RR	Risk Ratio
SBD	Sleep Breathing Disorder
SD	Standard Deviation
UIP	Usual Interstitial Pneumonia
FDA	Food and Drug Administration
VTE	Venous Thromboembolism
