# Supplementary material for: The Impact of Comorbidities on the Discontinuation of Antifibrotic Therapy in Patients with Idiopathic Pulmonary Fibrosis
Source: Pharmaceuticals (Basel). 2025 Mar 14;18(3):411. doi: 10.3390/ph18030411 (PMC11944575; doi:10.3390/ph18030411)
Supplement: Supplementary file 1 [file pharmaceuticals-18-00411-s001.zip › pharmaceuticals-3467314-supplementary.pdf]

pharmaceuticals

MDPI

## Supplementary Material

**Table S1.** Correlation between baseline characteristics and treatment suspension during the first year in the entire cohort and according to the anti-fibrotic drug.

| | Overall n=101 | p |
|---|---|---|
| Anti-fibrotic treatment | | |
| Pirfenidone | 6 (12.5) | 0.051 |
| Nintedanib | 15 (28.3) | |
| Age, median (IQR) | | |
| Suspended treatment | 75.0 (9.0) | 0.327 |
| Non-suspended treatment | 72 (11.3) | |
| Sex | | |
| Female | 8 (20.5) | 0.956 |
| Male | 13 (21.0) | |
| Comorbidities | | |
| <3 | 16 (21.6) | 0.734 |
| ≥3 | 5 (18.5) | |
| Respiratory comorbidity | | |
| No | 17 (22.1) | 0.568 |
| Yes | 4 (16.7) | |
| Non-respiratory comorbidity | | |
| No | 6 (23.1) | 0.739 |
| Yes | 15 (20.0) | |

Data are presented as No. (%), unless otherwise stated.
